# Data on spatial and temporal modelling of soil water storage in the Guinea savannah zone of Northern Ghana

**DOI:** 10.1016/j.dib.2022.108192

**Published:** 2022-04-19

**Authors:** Kwabena Abrefa Nketia, Stephen Boahen Asabere, Daniela Sauer

**Affiliations:** a Department of Physical Geography, University of Göttingen, Goldschmidtstr. 5, Göttingen 37077, Germany; b Council for Scientific and Industrial Research-Soil Research Institute, Kumasi, Ghana

**Keywords:** Guinea savannah, Semi-arid climate, Soil water storage, Spatio-temporal modelling, West Africa, Soil moisture, Predictive soil mapping, Machine learning

## Abstract

In this article, we present the space-time variability of soil moisture (SM) and soil water storage (SWS) from key agricultural benchmark soil types measured across the Guinea savannah zone of Ghana (*n* ≈ 2,000 measurements) in a single cropping season (Nketia et al., 2022). From 36 locations, SM measurements were obtained with a PR2/60 moisture probe calibrated for a 0–100 cm soil depth interval (at six depths). We further introduce a new pedotransfer model that was developed in deriving the SWS for the same depth interval of 0–100 cm. Assessing information on the space-time variability of SM and SWS is essential for agricultural intensification efforts, especially in semi-arid landscapes of sub-Saharan Africa (SSA), where there is the need and the potential to increase food-crop production. This dataset spans the main topographic units of the Guinea savannah zone and covers dominant vegetation types and land uses of the region, which is similar to most parts of West Africa. The comprehensive dataset and the customized machine learning models can be used to support crop production with respect to water management and optimized agricultural resource allocation in the Guinea savannah landscapes of Ghana and other parts of SSA.

## Specifications Table

**Table:** 

Subject	Agricultural Sciences.
Specific subject area	Soil science for sustainable agriculture, food security and soil water management.
Type of data	Tables, Figures, and ‘R’ data files.
How data were acquired	Station measurements of soil moisture (SM) using a probe.
Data format	Raw, analyzed.
Description of data collection	*In situ* SM content was taken at 36 locations across key agricultural benchmark soil locations using a calibrated Delta-T PR2/60 moisture probe at 0–100 cm soil depth (subdivided into six depths) in the Guinea savannah landscapes of Ghana. Sampling locations were stratified using a hybrid model, which coupled a global weighted principal component algorithm and a cost-constrained conditioned Latin hypercube algorithm.
Data source location	• Region: Northern Ghana (Tamale region), Guinea savannah agroecological zone. • Country: Ghana. • 36 Geographical Position System (GPS) coordinates of the measurement locations are included in the article.
Data accessibility	Data available within the article and also hosted on an open-access cloud repository: • Repository name: Zenodo • Data identification number (doi): 10.5281/zenodo.6447871 • Direct URL to data: https://zenodo.org/record/6447871#.YlQpc8hByHs.
Related research article	K.A. Nketia, S.B. Asabere, A. Ramcharan, S. Herbold, S. Erasmi, D. Sauer, Spatio-temporal mapping of soil water storage in a semi-arid landscape of Northern Ghana–A multi-tasked ensemble machine-learning approach, Geoderma. 410 (2022) 115,691. https://doi.org/10.1016/j.geoderma.2021.115691

## Value of the Data

• The data provides information on space-time SM and SWS over 0–100 cm soil depth for key agricultural benchmark soils of the Guinea savannah zone of Ghana.
• It provides useable information on the 4D SWS distribution of the Guinea savannah region of Ghana, which can support farmers in estimating where, when, how much, and for how long SWS is available for cultivation [1].
• The data is useful for soil and agronomic research into crop yield production limited by water stress, such as modelling scenarios of water management for dry-season farming.

## 1 Data Description

**Fig. 1:**
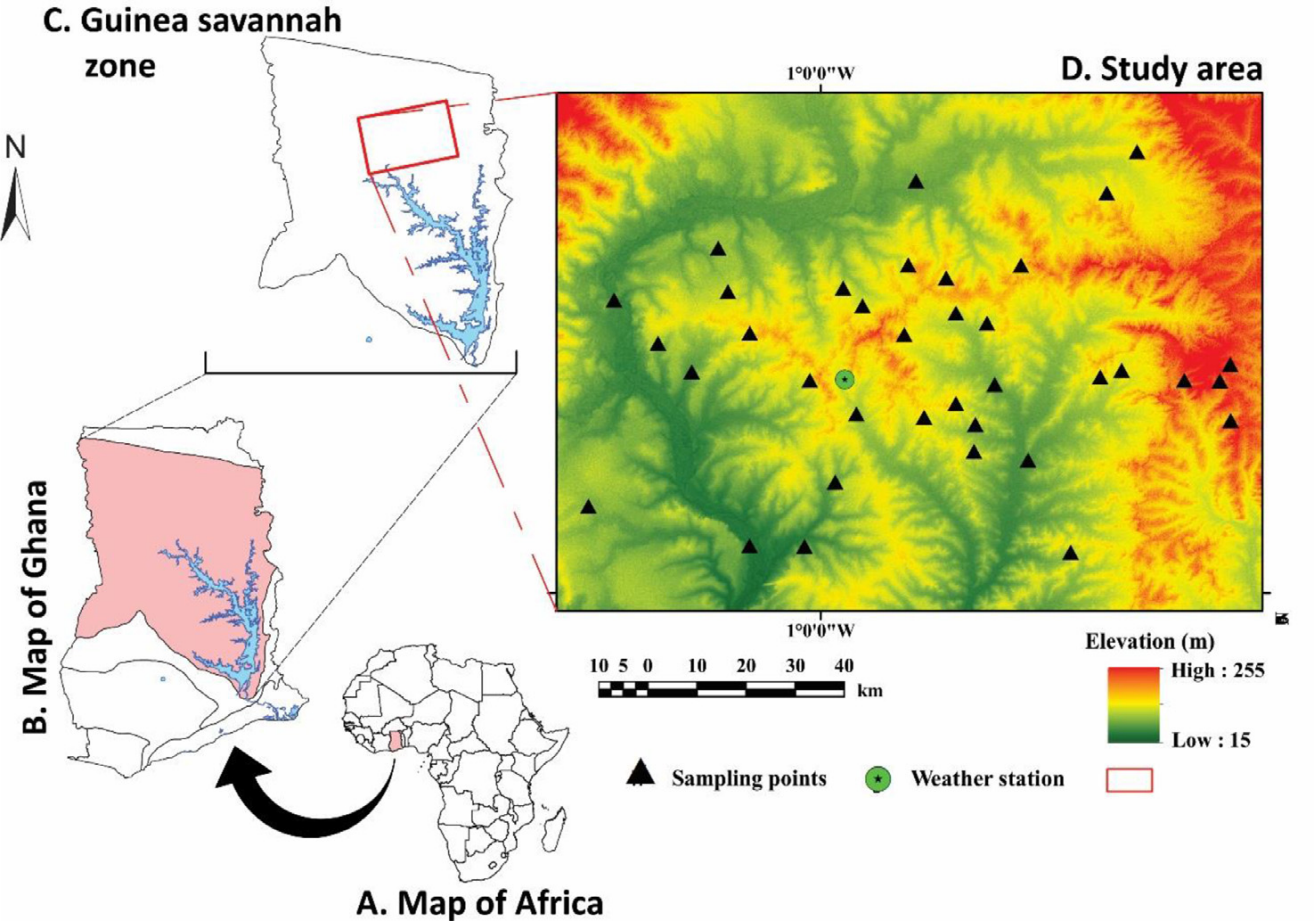
Map of Africa (A) showing study area (B, C) and *in situ* measurement locations (D), which are superimposed on the SRTM-DEM of the study area. Modified from Nketia et al. [3].

**Fig. 2:**
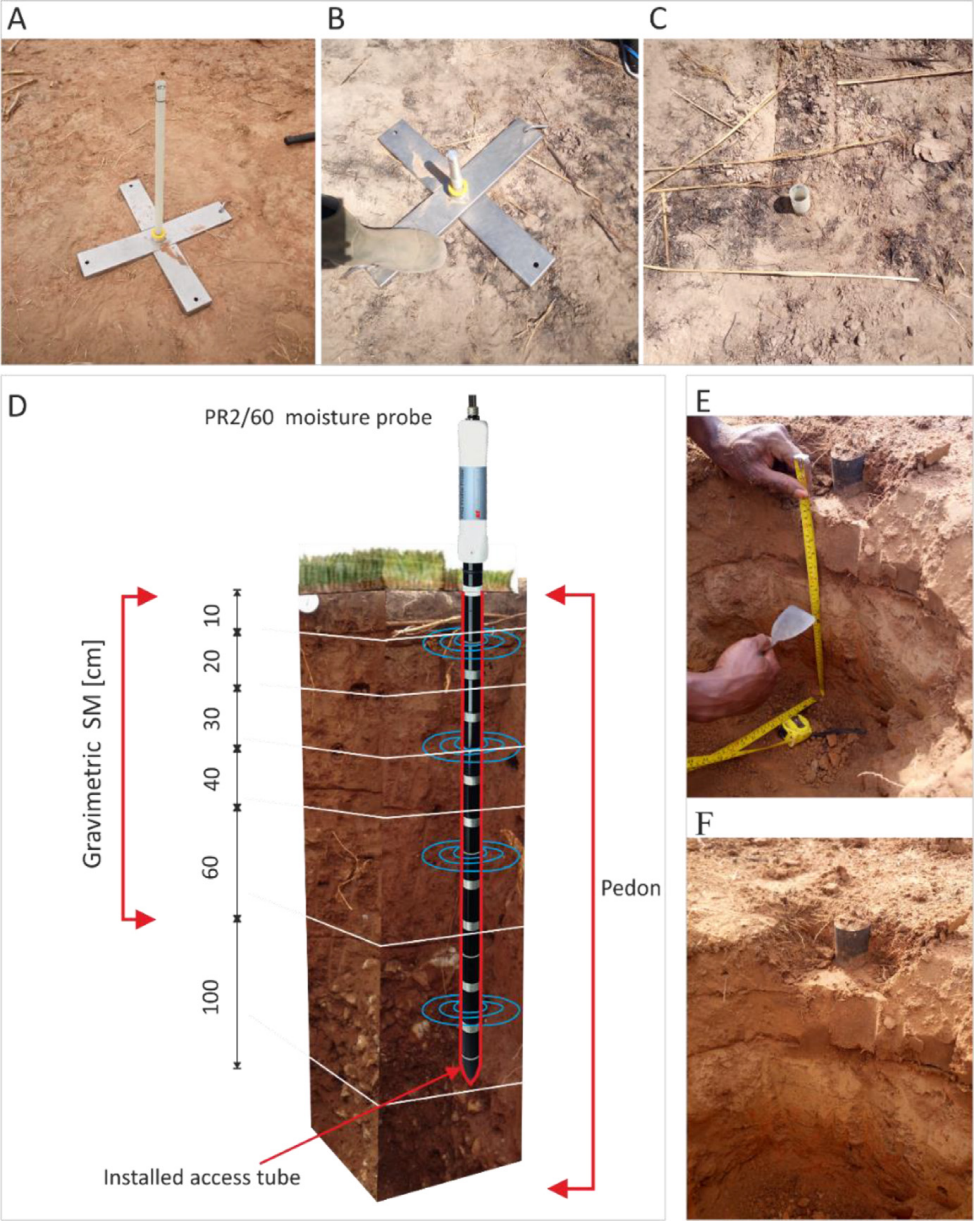
*In situ* SM measurement and undisturbed soil sampling. (A)–(C) Installation of access tubes for the PR2/60 moisture probes (Delta‐T Devices) down to a depth of 100 cm. (D) 3D representation of a soil profile with installed access tube and PR2/60 moisture probe. (E)–(F) Soil sampling with a stainless-steel cylinder at specific soil depths.

The data presented in this paper illustrates the space-time variability of soil moisture (SM) and soil water storage (SWS) of 36 stratified locations of the Guinea savannah zone of Ghana (*n* ≈ 2000). Fig. 1 shows the study area and locations from where *in situ* SM measurement were collected, covering a 170 × 190 km area across seven key agricultural benchmark soil types. Table 1 shows site characteristics and their associated GPS coordinates for the 36 measurement locations. Fig. 2 illustrates how the *in situ* SM measurements and soil sampling at each measurement location was conducted. We modelled vertical variation in SWS for the 36 locations, using a set of pedotransfer algorithms, converting the *in situ* measured SM at standard depths (i.e., 10, 20, 30, 40, 60, and 100 cm) into six depth intervals (i.e., 0–5, 5–15, 15–30, 30–40, 40–60, and 60–100 cm) as per *GlobalSoilMap* specifications [2]. The data file called ‘Code_C1.R’ (https://zenodo.org/record/6447871#.YlQpc8hByHs) shows the fully commented systematic SWS modelling framework used in deriving SWS data within this article.

**Table 1:** Site characteristics and GPS coordinates for all sampling locations from the Guinea savannah zone of Ghana.

Station ID	Latitude **[°]**	Longitude **[°]**	Soil type⁎⁎	Soil association⁎⁎	WRB classification	Geology	District block
*Sites where SM and physical soil properties were determined*							
AT01	9.38209	−0.68264	Lima	Sambu-Pasga	Gleyic Planosols	Shale, Mudstone, Sandstone	Mion
AT02	9.3898	−1.02133	Kpelesawgu	Sambu-Pasga	Eutric Plinthosols	Shale, Mudstone, Sandstone	Tolon
AT03	9.24358	−0.62165	Changnalili	Lima-Volta	Petric Plinthosols	Alluvial sediments	Karaga
AT04	9.30885	−0.71828	Kpelesawgu	Techiman-Tampu	Eutric Plinthosols	Voltain sandstone	Tamale Metro
AT05	9.40523	−1.23727	Changnalili	Kpelesawgu-Changnalili	Petric Plinthosols	Voltain shale	Tolon
AT06	9.55798	−0.96041	Lima	Lima-Volta	Gleyic Planosols	Alluvial sediments	Kumbungu
AT07	9.55211	−1.17127	Lima	Sambu-Pasga	Gleyic Planosols	Shale, Mudstone, Sandstone	Tolon
AT08	9.34742	−0.75396	Lima	Techiman-Tampu	Gleyic Planosols	Voltain sandstone	Tamale Metro
AT09	9.2598	−0.72064	Lima	Lima-Volta	Gleyic Planosols	Alluvial sediments	East Gonja
AT10	9.45722	−1.29907	Lima	Kpelesawgu-Changnalili	Gleyic Planosols	Voltain shale	North Gonja

*Sites where in situ measurements were taken*							
AT11	9.63135	−1.18874	Lima	Kpelesawgu-Changnalili	Gleyic Planosols	Voltain shale	Kumbungu
AT12	9.39602	−0.48972	Kumayili	Techiman-Tampu	Chromic Lixisols	Voltain sandstone	Tamale Metro
AT13	9.41191	−0.23344	Changnalili	Gushiagu-kasele	Petric Plinthosols	Voltain shale	Karaga
AT15	9.32232	−0.81182	Lima	Lima- Volta	Gleyic Planosols	Alluvial sediments	East Gonja
AT16	9.08735	−1.13139	Siare	Siare-dagare	Plinthic Lixisols	Alluvial sediments	Central Gonja
AT17	9.80794	−0.4222	Lima	Kpelesawgu-Changnalili	Gleyic Planosols	Voltain shale	Karaga
AT18	9.08612	−1.03072	Volta	Kpelesawgu-Changnalili	Fluvic Gleysols	Voltain shale	Central Gonja
AT19	9.57707	−0.7718	Lima	Techiman-Tampu	Gleyic Planosols	Voltain sandstone	Savelugu Nanton
AT20	9.31643	−0.25106	Lima	Sambu-Pasga	Gleyic Planosols	Shale, Mudstone, Sandstone	Mion
AT22	9.75427	−0.82686	Volta	Lima-Volta	Fluvic Gleysols	Alluvial sediments	Kumbungu
*Sites where in situ measurements were taken*							
AT23	9.51278	−0.75376	Lima	Lima-Volta	Gleyic Planosols	Alluvial sediments	Savelugu Nanton
AT24	9.47611	−1.13107	Kumayili	Sambu-Pasga	Chromic Lixisols	Shale, Mudstone, Sandstone	Savelugu Nanton
AT25	9.38885	−0.2706	Kpelesawgu	Techiman-Tampu	Eutric Plinthosols	Voltain sandstone	Tamale Metro
AT26	9.53645	−1.37973	Dagare	Sambu-Pasga	Gleyic Fluvisols	Shale, Mudstone, Sandstone	North Gonja
AT27	9.52581	−0.92455	Lima	Lima-Volta	Gleyic Planosols	Alluvial sediments	Kumbungu
AT28	9.73252	−0.47761	Lima	Sambu-Pasga	Gleyic Planosols	Shale, Mudstone, Sandstone	Karaga
AT29	9.15954	−1.42628	Lima	Techiman-Tampu	Gleyic Planosols	Voltain sandstone	Central Gonja
AT30	9.40799	−0.45041	Lima	Lima-Volta	Gleyic Planosols	Alluvial sediments	Mion

*Sites where in situ measurements were taken*							
AT31	9.07524	−0.54386	Lima	Sambu-Pasga	Gleyic Planosols	Shale, Mudstone, Sandstone	East Gonja
AT32	9.32838	−0.93585	Lima	Lima-Volta	Gleyic Planosols	Alluvial sediments	Central Gonja
AT33	9.60072	−0.84122	Kumayili	Mimi-Techiman	Chromic Lixisols	Voltain sandstone	North Gonja
AT34	9.59978	−0.63474	Lima	Kpelesawgu-Changnalili	Gleyic Planosols	Voltain shale	Savelugu Nanton
AT35	9.38973	−0.33578	Lima	Lima-Volta	Gleyic Planosols	Alluvial sediments	Mion
AT36	9.49393	−0.69697	Lima	Lima-Volta	Gleyic Planosols	Alluvial sediments	Karaga
AT37	9.47358	−0.84833	Lima	Techiman-Tampu	Gleyic Planosols	Voltain sandstone	Sagnerigu
AT38	9.20374	−0.97482	Volta	Kpelesawgu-Changnalili	Fluvic Gleysols	Voltain shale	Central Gonja

*Sites for plant available water content*							
Wet 1	9.65884	−0.57731	Lima	Lima-Volta	Gleyic Planosols	Alluvial sediments	Karaga
Wet 2	9.40714	−0.98608	Kpelesawgu	Sambu-Pasga	Eutric Plinthosols	Shale, Mudstone, Sandstone	Tolon

⁎⁎ according to the Ghanaian soil classification system.

**Fig. 3:**
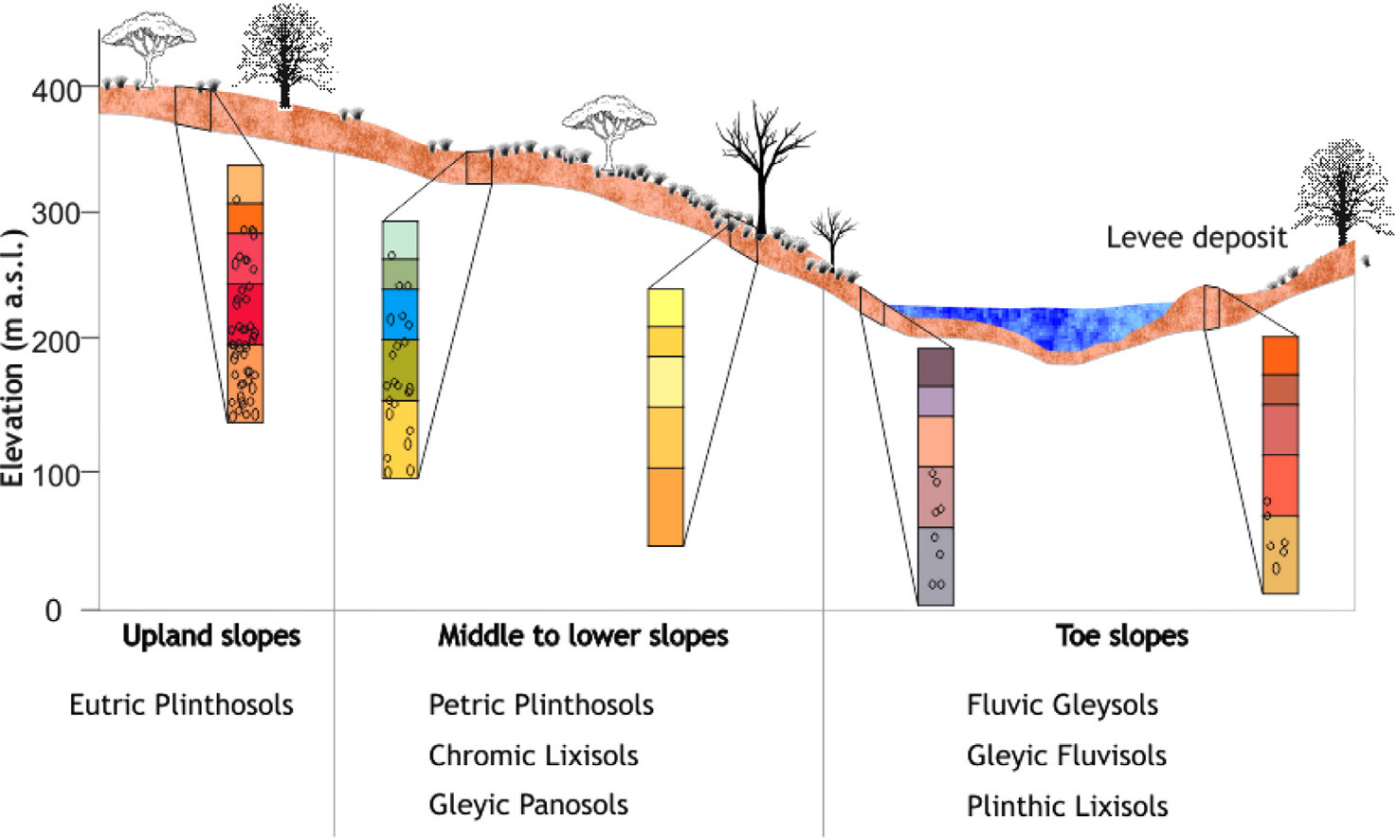
Soil types along the three topographical units. Chart not drawn to scale. Modified from Nketia et al. [1].

Fig. 3 depicts a soil catena showing the soil types of the study area along which measurements were undertaken. The dataset can also be grouped based on the seven key benchmark soil types covering the three topographical units of the study area (Fig. 3) [4]. The upper slope is covered by Eutric Plinthosols (Kpelesawgu series in the local Ghanaian soil classification system). Soils on middle to lower slopes include Gleyic Planosols (Lima series), Petric Plinthosols (Changnalili series) and Chromic Lixisols (Kumayili series), and soils on toe slopes are Gleyic Fluvisols (Dagare series), Plinthic Lixisols (Siare series) and Fluvic Gleysols (Volta series).

The shared dataset reported in the article is stored in an excel file, called ‘File_T1_SM_SWS.xlsx’ (https://zenodo.org/record/6447871#.YlQpc8hByHs). The ‘File_T1_SM_SWS.xlsx’ file contains two spreadsheets, i.e*.*, ‘SM’ for raw SM data, and ‘SWS’ for the calculated SWS data. The variables in each data sheet are specified below:

• Sheet ‘SM’ shows the station IDs of the *in situ* measurement locations (column 1) and the lower soil depths (in cm) at which SM measurements were taken (column 2). Columns 3 and 4 contain the raw volumetric SM measurements expressed in percentages and their associated measurement dates, respectively. Columns 5 and 6 show WGS84 coordinates of the measurement stations in latitude and longitude, respectively.
• In sheet ‘SWS’, also station IDs of the measurement locations (column 1) and their corresponding dates of measurement (column 2) are presented. Columns 3 and 4 contains the upper and lower soil depth, respectively, (both in cm), for which SM measurements were taken. Columns 5 and 6 contains the benchmark soil types (in the Ghanaian local system) and their equivalent FAO World Reference Base classification, respectively. Columns 7 and 8 show soil thickness (in cm) and its corresponding calculated SWS (expressed by an absolute value in mm), respectively. Column 9 shows the topographic units along which the seven key benchmark soil types occur.

The data covers soils under different vegetation types such as Borassus palm (*Borassus aethiopum)*, Senegal mahogany (*Khaya senegalensis*), shea tree (*Vitellaria paradoxa)* and natural grassland (*Pennisetum purpureum*), and soils under various types of land use, including dryland farming, irrigated vegetable cultivation and pastures.

## 2 Experimental Design, Materials and Methods

### 2.1 *In situ* SM measurements

The 36 measurement locations were stratified following an unbiased approach that coupled the global weighted principal component algorithm with a cost-constrained conditioned Latin hypercube algorithm [3]. With this approach, it was possible to account for the maximum local spatial structures of the study area, while selecting optimized locations that highly influenced SM variability.

SM measurements were taken in 36 soil profiles, located on the three main soil topographic units: upper, middle-lower, and toe slopes (Fig. 3). At each location, an access tube was installed (Fig. 2A–C), where SM was measured at six standard depths within the 0–100 cm depth (i.e., 10, 20, 30, 40, 60, and 100 cm) using a calibrated moisture probe (PR2/60, Delta-T Devices) (Fig. 2D). One of the objectives of work reported in the associated paper of this data article, Nketia et al. [1] was to estimate SM from Sentinel-1 data. Thus, the SM measurements were timed to coincide with the overpass of the Sentinel-1 satellite at a temporal resolution of 12 days for ten time-steps covering the whole dry season (i.e., February–June). Thus, in total 2,160 soil measurements were taken.

### 2.2 SWS modelling framework

**Fig. 4:**
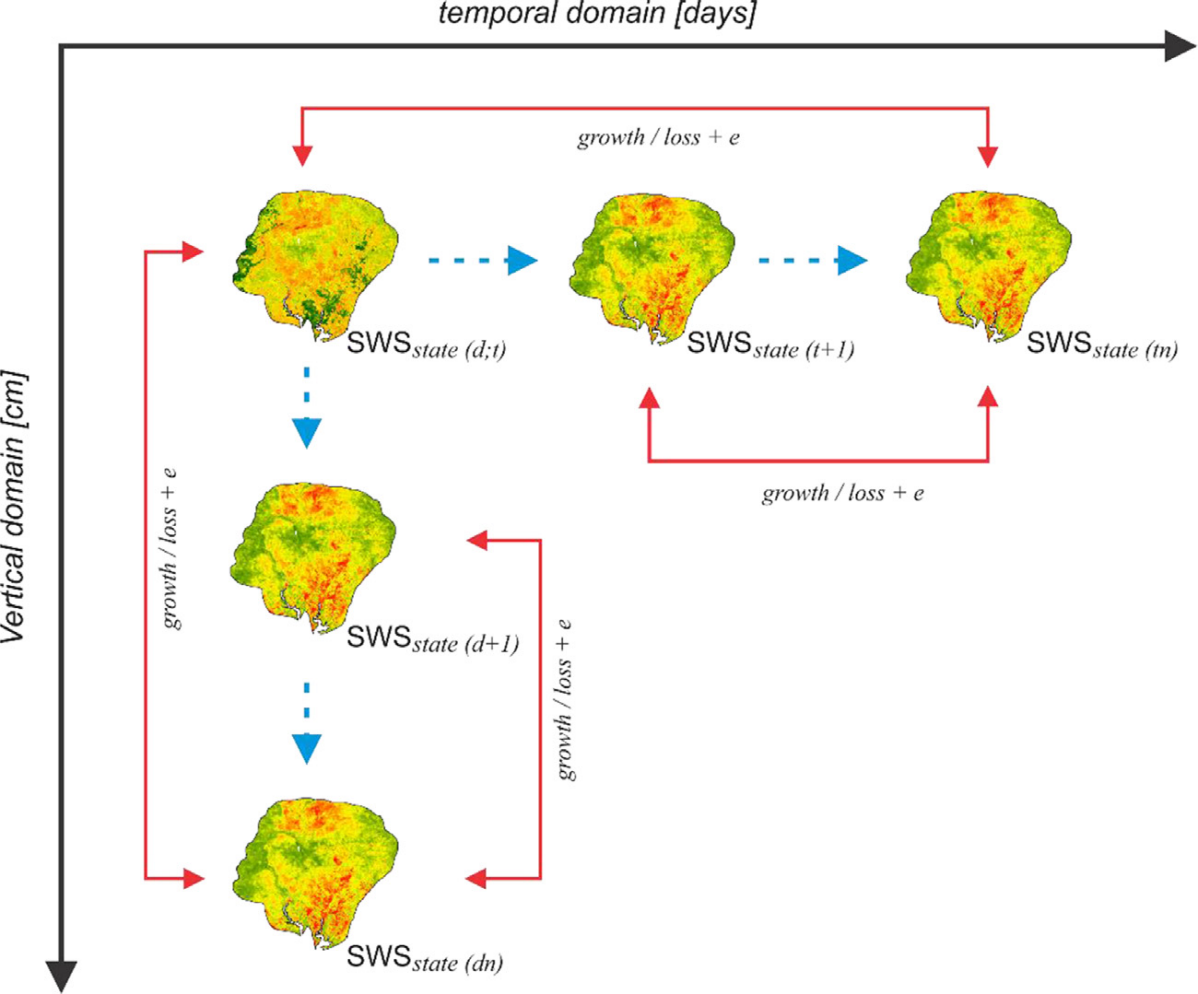
Space-time function for estimating SWS at a location for defined soil-depth intervals. Modified from Nketia et al. [1].

An important contribution of this data is the modelled SWS. This part of the data was derived by implementing a pedotransfer algorithm in two main stages as illustrated in Fig. 4. In a first step, *in situ* SM measurements were vertically discretized into six depth intervals (i.e., 0–5, 5–15, 15–30, 30–40, 40–60, and 60–100 cm) following the *GlobalSoilMap* specifications [2]. In a second step, SWS at each data point was recursively profiled as a function of the measured *in situ* SM, bulk density and the effective soil thickness between two soil layers [1]. By this approach, we accounted for the differential availability of SWS critical to the management of shallow and deep-rooted plants notable to the study area. This approach also allowed us to account for the effect of soil depth on *in situ* SM measurements.

**Fig. 5:**
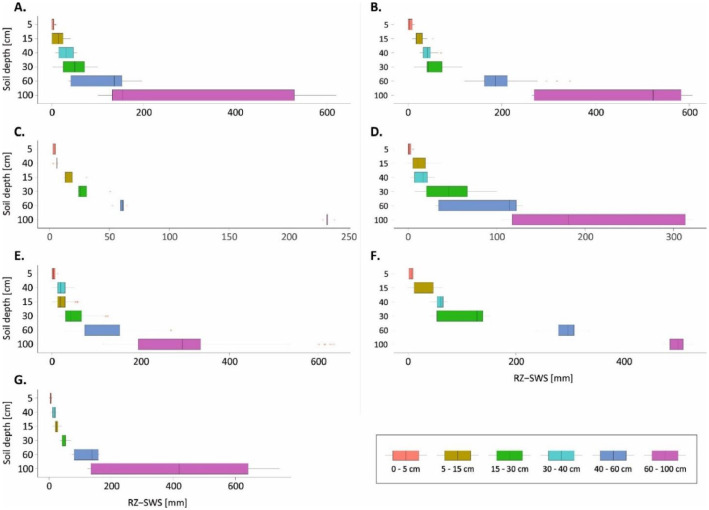
Space-time variability of SWS for benchmark soil types; (A) Kpelesawgu series, (B) Changnalili series, (C) Dagare series, (D) Kumayili series, (E) Lima series, (F) Siare series and (G) Volta series along the various *in situ* measurement depths. Soil names are in Ghanaian soil classification system. Statistical measures (range – length of whiskers and medians – vertical bars) indicating the space-time variability of SWS are also shown. RZ-SWS means rootzone soil water storage.

The study area is characterized by an inherent plinthic and petro-plinthic horizon, occurring at 70–100 cm depth [4] and thus restricting water movements between lower and upper soil layers. Because of this situation groundwater movement was not considered in the SWS modelling framework. Thus, we only assumed SWS for the succeeding soil depth (d) as a reservoir for the preceding soil depth (d−1) at a time-step (t). For this rationale, observed changes in measured SM of the soil depths is proportional to the change in modelled SWS at a location between a preceding and a succeeding soil depth at a time-step. Eq. (1) defines the SWS model, which is expressed by an absolute value in mm. For each *in situ* SM measurement at each point in time and soil depth, we accounted for the SWS loss or gain at this point with respect to its initial state [5,6]. Well annotated R [7] scripts that were used in modelling SWS are presented in the file ‘Code_C1.R’ (available at https://zenodo.org/record/6447871#.YlQpc8hByHs).

(1) $$SWS_{itd}=0.1*f{\left(SM_{itd},BD_{id},h_{id}\right)}_{t}+R_{t}*f{\left(SM_{id},BD_{id},h_{id}\right)}_{t-1}$$

where input parameters for function (f), calculated at a constant factor of 0.1 (from density of water of 1 g cm^-3^), were *in situ* SM $\left(SM_{itd};\%Vol\right)$ at location (i), time-step (t) and soil depth (d), bulk density laboratory data $\left(BD_{id};g\;cm^{-3}\right)$ and respective soil thickness $\left(h_{\mathrm{id}};\mathrm{cm}\right)$. $R_{t}$ explains the rate of loss or gain in $SM_{itd}$ between a preceding and subsequent soil depth interval [6], and varies from 0 (low loss or gain) to ± 1 (high loss or gain). Fig. 5 illustrates the variability of SWS per each benchmark soil type along the *in situ* measurement depth intervals.

## Ethics Statement

There is no conflict of interest. The data is available to the general public.

## Supplementary Material

Full datasets (i.e., Code_C1.R, File_T1_SM_SWS.xlsx and ‘Spatio-temporal variability of SWS.pdf’) are hosted on an open-access repository at https://zenodo.org/record/6447871#.YlQpc8hByHs.

## CRedit Author Statement

**Kwabena Abrefa Nketia:** Conceptualization, Data curation, Methodology, Investigation, Visualization, Writing – original draft; **Stephen Boahen Asabere:** Conceptualization, Methodology, Investigation, Writing – review & editing; **Daniela Sauer:** Consultation/support in the conceptualization and in the realization of the methodological approach, Writing – review & editing.

## Declaration of Competing Interest

The authors declare that they have no known competing financial interests or personal relationships that could have appeared to influence the work reported in this paper.

## Data Availability

Data on space-time soil moisture and modelled soil water storage, Ghana (Original data) (Zenodo).
